# Continuous Blood Pressure Monitoring in Patients Having Surgery: A Narrative Review

**DOI:** 10.3390/medicina59071299

**Published:** 2023-07-14

**Authors:** Alina Bergholz, Gillis Greiwe, Karim Kouz, Bernd Saugel

**Affiliations:** 1 Department of Anesthesiology, Center of Anesthesiology and Intensive Care Medicine, University Medical Center Hamburg-Eppendorf, 20251 Hamburg, Germany; a.bergholz@uke.de (A.B.); g.greiwe@uke.de (G.G.); karim.kouz@gmail.com (K.K.); 2 Outcomes Research Consortium, Cleveland, OH 44195, USA

**Keywords:** anesthesia, arterial pressure, cardiovascular dynamics, hemodynamic monitoring, intraoperative hypotension

## Abstract

Hypotension can occur before, during, and after surgery and is associated with postoperative complications. Anesthesiologists should thus avoid profound and prolonged hypotension. A crucial part of avoiding hypotension is accurate and tight blood pressure monitoring. In this narrative review, we briefly describe methods for continuous blood pressure monitoring, discuss current evidence for continuous blood pressure monitoring in patients having surgery to reduce perioperative hypotension, and expand on future directions and innovations in this field. In summary, continuous blood pressure monitoring with arterial catheters or noninvasive sensors enables clinicians to detect and treat hypotension immediately. Furthermore, advanced hemodynamic monitoring technologies and artificial intelligence—in combination with continuous blood pressure monitoring—may help clinicians identify underlying causes of hypotension or even predict hypotension before it occurs.

## 1. Introduction

In patients having surgery under general anesthesia, profound and prolonged hypotension is associated with organ injury—specifically, acute kidney and myocardial injury [1,2]—and postoperative mortality [3,4,5,6]. Hypotension can occur after anesthetic induction before surgical incision (postinduction hypotension), during surgery (intraoperative hypotension), and after surgery (postoperative hypotension) [7,8,9]. Associations with organ injury have been shown for postinduction hypotension [8], intraoperative hypotension [1,2,3,4,5], and postoperative hypotension [9,10,11,12].

While the association between perioperative hypotension and organ injury is well established, optimal blood pressure targets for individual patients remain controversial [13,14,15]. Pending results from robust clinical trials on targeted blood pressure management [16] (ClinicalTrials.gov identifier: NCT04884802), anesthesiologists should avoid profound and prolonged hypotension and maintain mean arterial pressure (MAP) above 65 mmHg during surgery [17]—and presumably somewhat higher postoperatively [6,18].

A crucial part of avoiding hypotension is accurate and tight blood pressure monitoring. Improving blood pressure monitoring to help avoid perioperative hypotension is thus a matter of ongoing research. Continuous blood pressure monitoring with arterial catheters or noninvasive sensors provides blood pressure in real-time and may enable clinicians to detect and treat hypotension immediately. Furthermore, advanced hemodynamic monitoring technologies and predictive monitoring may help clinicians identify underlying causes of hypotension or even predict hypotension before it occurs [19,20].

In this article, we briefly describe methods for continuous blood pressure monitoring, discuss current evidence for continuous blood pressure monitoring in patients having surgery to reduce perioperative hypotension, and expand on future directions and innovations in this field.

## 2. Methods for Continuous Blood Pressure Monitoring

The clinical reference method (i.e., gold standard) to continuously measure blood pressure is intraarterial blood pressure monitoring with an arterial catheter [21]. However, intraarterial blood pressure monitoring requires the cannulation of an artery, which can very rarely cause serious complications such as ischemia or major bleeding [22].

Noninvasive finger sensors allow blood pressure to be monitored continuously without arterial cannulation. Noninvasive blood pressure monitoring with finger sensors is based on the vascular unloading technique [23,24]. The underlying measurement principle is based on the theory that a force exerted by a body can be determined by measuring a counterforce – assuming a steady physical coupling [25]. Finger sensors contain an inflatable finger cuff and integrated infrared photodiodes and light detectors. The infrared system measures the blood volume in the finger arteries which changes periodically throughout the cardiac cycle. A control system adjusts the cuff pressure high-frequently to keep the blood volume in the finger arteries constant. Based on the cuff pressure adjustments, a continuous blood pressure waveform can be reconstructed and analyzed to obtain absolute blood pressure values. Validation studies investigating the accuracy of finger sensor-derived blood pressures compared to intraarterial blood pressures revealed heterogeneous results [26]. On the one hand, several studies demonstrated interchangeability between finger sensor and intraarterial blood pressure monitoring [26,27,28,29,30] and—even more importantly—superiority of finger sensor blood pressure monitoring compared to oscillometric blood pressure monitoring [31]. For example, in patients having bariatric surgery, the agreement between finger sensor and intraarterial blood pressures was better than the agreement between oscillometric and intraarterial blood pressures [31]. On the other hand, there are also studies suggesting that finger sensor and intraarterial blood pressure monitoring are not interchangeable [32,33,34,35]. Finger sensor blood pressure monitoring may thus be used instead of intermittent oscillometric blood pressure monitoring rather than instead of intraarterial blood pressure monitoring. Finger sensor blood pressure monitoring becomes unreliable in patients with impaired finger perfusion—such as patients with circulatory shock or high-dose vasopressor therapy [28,36]—who usually have intraarterial monitoring anyway.

## 3. Continuous Blood Pressure Monitoring during Anesthetic Induction

Postinduction hypotension, i.e., hypotension between the start of anesthetic induction and surgical incision, is common [7,37,38] and associated with acute kidney injury [8]. Postinduction hypotension accounts for about one-third of all perioperative hypotension [8] and is mainly determined by anesthetic management [39]—specifically vasodilation by anesthetic drugs [38]. Postinduction hypotension is “scheduled hypotension” that usually rapidly occurs after the administration of anesthetic drugs.

Despite the obvious cause of postinduction hypotension, blood pressure is usually monitored only intermittently using upper-arm cuff oscillometry during anesthetic induction even if intraarterial blood pressure monitoring with an arterial catheter is planned for intraoperative blood pressure monitoring. In a recent survey, almost two-thirds of European anesthesiologists stated that they would insert arterial catheters usually only after the administration of anesthetic induction agents, i.e., when the patient is already under general anesthesia—instead of inserting arterial catheters before anesthetic induction [40]. It is reasonable to assume that intermittent blood pressure monitoring easily misses hypotension and that continuous—compared to intermittent—blood pressure monitoring may reduce postinduction hypotension.

A single-center randomized trial including 242 noncardiac surgery patients indeed confirmed that continuous intraarterial compared to intermittent oscillometric blood pressure monitoring reduces hypotension during anesthetic induction [41]. All patients had a clinical indication for intraarterial blood pressure monitoring during surgery. In all patients, the arterial catheter was inserted before anesthetic induction. Patients were then assigned to continuous intraarterial or intermittent oscillometric blood pressure monitoring (with blinded continuous intraarterial blood pressure monitoring) during the first 15 min of anesthetic induction. The median (25th percentile, 75th percentile) area under a MAP of 65 mmHg was 46 (7, 111) mmHg x min in patients assigned to intermittent oscillometric blood pressure monitoring and 15 (2, 36) mmHg x min in patients assigned to continuous intraarterial blood pressure monitoring. Patients assigned to intermittent oscillometric blood pressure monitoring spend a median duration of 5.4 (2.1, 9.5) min below a MAP of 65 mmHg—compared to 2.6 (0.7, 5.0) min in patients assigned to continuous intraarterial blood pressure monitoring [41]. In this trial, continuous intraarterial blood pressure monitoring thus not only reduced the duration but also the severity of hypotension [41]. In patients for whom continuous intraarterial blood pressure monitoring is planned, clinicians should thus consider inserting arterial catheters before, rather than after, anesthetic induction.

Most patients having surgery do not have a clinical indication for intraarterial blood pressure monitoring with an arterial catheter [42]. In these patients, who are monitored with intermittent oscillometry in today’s clinical practice, finger sensors could be used for continuous blood pressure monitoring. The decision whether to use arterial catheters or finger sensors depends on patient- and surgery-related risk factors [6].

Whether finger sensor blood pressure monitoring helps reduce postinduction hypotension is a subject of current research. In a single-center randomized trial with 242 noncardiac surgery patients, patients were assigned to continuous finger sensor blood pressure monitoring or intermittent oscillometric blood pressure monitoring (with blinded continuous finger sensor blood pressure monitoring) [43]. Continuous finger sensor—compared to intermittent oscillometric—blood pressure monitoring, reduced the severity and duration of hypotension during the first 15 min of anesthetic induction. Based on the results of this trial, clinicians should consider continuous finger sensor blood pressure monitoring rather than intermittent oscillometric blood pressure monitoring during anesthetic induction.

These trials suggest that continuous blood pressure monitoring during anesthetic induction reduces hypotension compared to intermittent blood pressure monitoring. To reduce postinduction hypotension, blood pressure should thus be monitored continuously during anesthetic induction, either with an arterial catheter or with a finger sensor. However, the extent of hypotension reduction on postoperative complications is unclear and needs to be investigated in large randomized trials.

## 4. Continuous Blood Pressure Monitoring during Surgery

Intraoperative hypotension, i.e., hypotension during surgery, is common [44] and associated with acute kidney injury, acute myocardial injury, and mortality [1,45,46]. Several trials investigated the effect of continuous blood pressure monitoring on intraoperative hypotension [43,47,48,49].

In a randomized trial, 306 patients having noncardiac surgery were assigned either to continuous intraarterial blood pressure monitoring or intermittent oscillometric blood pressure monitoring [50]. Hypotension was quantified either based on continuous intraarterial or intermittent oscillometric blood pressure monitoring. The median area under a MAP of 65 mmHg was 24 (1, 121) mmHg x min in patients assigned to continuous intraarterial blood pressure monitoring and 10 (0, 57) mmHg x min in patients assigned to intermittent oscillometric blood pressure monitoring. This trial shows that continuous intraarterial blood pressure monitoring detects more than twice as much hypotension as intermittent oscillometric blood pressure monitoring [50]. These results may be in part explainable by the intermittent measurement character and overestimation of low blood pressures by oscillometry [51].

A trial including 316 moderate-to-high-risk patients investigated whether continuous finger sensor blood pressure monitoring reduces intraoperative hypotension compared to intermittent oscillometric blood pressure monitoring (with blinded finger sensor blood pressure monitoring) [49]. Hypotension was quantified using the time-weighted average MAP < 65 mmHg. The median time-weighted average MAP < 65 mmHg was 0.05 (0.00, 0.22) mmHg in patients assigned to continuous finger sensor blood pressure monitoring and 0.11 (0.00, 0.54) mmHg in patients assigned to intermittent oscillometric blood pressure monitoring.

These results are in line with a trial in 160 patients having orthopedic surgery under general anesthesia in which patients were assigned to continuous finger sensor blood pressure monitoring or to intermittent oscillometric blood pressure monitoring [48]. Hypotension was defined as a single MAP measurement < 60 mmHg. Hypotension occurred less frequently in patients assigned to continuous finger sensor blood pressure monitoring than in patients assigned to intermittent oscillometric blood pressure monitoring (51 hypotensive events *versus* 19 hypotensive events).

In another randomized trial, 242 noncardiac surgery patients were assigned to continuous finger sensor blood pressure monitoring or to intermittent oscillometric blood pressure monitoring (with blinded continuous finger sensor blood pressure monitoring) [43]. The severity and duration of hypotension during surgery quantified as the time-weighted average MAP < 65 mmHg was lower in patients assigned to continuous finger sensor blood pressure monitoring compared to patients assigned to intermittent oscillometric blood pressure monitoring [43].

## 5. Continuous Blood Pressure Monitoring after Surgery

Postoperative hypotension, i.e., hypotension within the first days after surgery, is common [52] and associated with acute kidney injury, myocardial injury, and mortality [9,10,11]. A substudy of the POISE-2 trial with more than 9000 patients showed that patients with postoperative hypotension within the first four postoperative days have almost three times higher odds of 30-day myocardial infarction and death than patients without postoperative hypotension [9]. Nearly one in five postoperative patients suffers from hypotension (defined as a MAP < 65 mmHg for at least 15 consecutive minutes) during the first two postoperative days [52]. On normal wards, blood pressure is usually only spot-checked every 4–8 h. Thus, postoperative hypotension often remains undetected and untreated [52,53,54]. For example, hypotensive episodes defined as a MAP < 70 mmHg for at least 15 consecutive minutes are not detected on normal wards in approximately 80% of all cases [53].

To detect postoperative hypotension, close or even continuous blood pressure monitoring would be needed [52,53]. As severe postoperative complications are often preceded by hypotension several minutes or hours earlier [55], timely recognition of hypotension may help improve patient outcomes.

In intensive care units, blood pressure is routinely measured continuously using arterial catheters. However, on normal wards, blood pressure is measured by nurses only every few hours. Continuous monitoring of blood pressure—and other vital signs—is an intriguing new concept that has the potential to revolutionize patient care [56,57,58,59]. Continuous blood pressure monitors on normal wards need to be small, noninvasive, and untethered. Additionally, they should not interfere with patients’ mobility or sleep. Different technologies for continuous blood pressure monitoring on normal wards are available, including the vascular unloading technique, pulse-wave transit time, and pulse decomposition [60,61,62]. Finger sensors can be used for continuous blood pressure monitoring on normal wards, but current systems still are not wireless and not well tolerated by patients [54]. Miniaturized wireless finger sensor systems are currently being developed [63]. Blood pressure sensors such as chest patches or wrist sensors are often photoplethysmography-based and use the pulse-wave transit time method to estimate blood pressure [61,64,65,66]. Several variables, including pulse rate and peripheral oxygen saturation, can be accurately monitored with wearable sensors. However, studies investigating the measurement performance of wearable sensors to measure blood pressure revealed contradicting results. Many of the currently available devices do not yet pass criteria of international measurement performance standards [67,68].

## 6. Will Artificial Intelligence Change Continuous Blood Pressure Monitoring?

**Figure 1:**
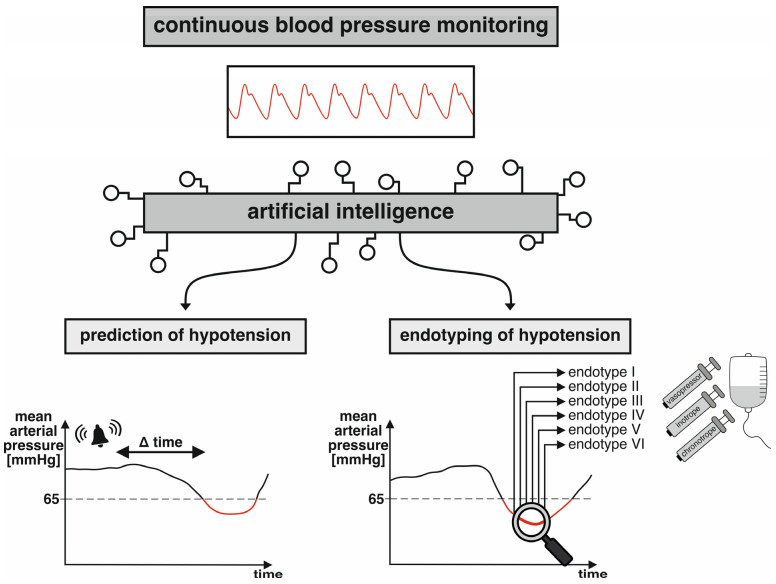
Artificial intelligence and continuous blood pressure monitoring. The figure shows two applications of artificial intelligence that can be used to predict hypotension or identify distinct endotypes of hypotension.

Artificial intelligence may substantially change current concepts of continuous blood pressure monitoring. Current blood pressure management is mainly reactive, with hypotension being treated only when it has already occurred [69]. Artificial intelligence can be used to analyze the blood pressure waveform and predict hypotension (Figure 1). Predictive monitoring may help avoid hypotension [69]. For example, the hypotension prediction index software (HPI-software) (Edwards Lifesciences, Irvine, CA, USA) analyzes the blood pressure waveform and provides a unitless number ranging from 0 to 100 that is an indicator for the likelihood of a patient developing hypotension [20]. The HPI-software was developed using a machine-learning algorithm considering waveform features [20]. HPI-software monitoring can be used on blood pressure waveforms from arterial catheters or finger sensors [70]. Trials investigating whether HPI-software monitoring reduces intraoperative hypotension compared to blood pressure management without HPI-software monitoring revealed contradictory results [71,72]. Robust trials are needed to investigate the effect of HPI-software monitoring on postoperative complications.

Artificial intelligence can also be used to identify distinct underlying causes of hypotension (Figure 1). In a recent study in patients having major abdominal surgery, an unsupervised machine-learning algorithm was used to identify endotypes of intraoperative hypotension based on essential hemodynamic variables, including stroke volume index, heart rate, cardiac index, systemic vascular resistance index, and pulse pressure variation [19]. Six different endotypes were identified: myocardial depression, bradycardia, vasodilation with cardiac index increase, vasodilation without cardiac index increase, hypovolemia, and mixed type [19]. Identifying and considering these different endotypes may help clinicians to treat hypotension causally and may help improve postoperative outcomes.

## 7. Conclusions

In patients having surgery under general anesthesia, profound and prolonged hypotension is associated with organ injury—specifically, acute kidney and myocardial injury—and postoperative mortality. Hypotension can occur after anesthetic induction before surgical incision (postinduction hypotension), during surgery (intraoperative hypotension), and after surgery (postoperative hypotension). Anesthesiologists should avoid profound and prolonged hypotension and maintain MAP above 65 mmHg during surgery—and presumably somewhat higher postoperatively. A crucial part of avoiding hypotension is accurate and tight blood pressure monitoring. The clinical reference method to continuously measure blood pressure is intraarterial blood pressure monitoring with an arterial catheter. Noninvasive finger sensors allow blood pressure to be monitored continuously without arterial cannulation. Trials suggest that continuous blood pressure monitoring during anesthetic induction and during surgery reduces hypotension compared to intermittent blood pressure monitoring. To reduce postinduction and intraoperative hypotension, blood pressure should thus be monitored continuously—either with an arterial catheter or with a finger sensor. However, the effect of hypotension reduction on postoperative complications is unclear and needs to be investigated in large randomized trials. Postoperative hypotension, i.e., hypotension within the first days after surgery, is common—and mainly missed by current spot-check measurements by nurses. Continuous monitoring of blood pressure is an intriguing new concept that has the potential to revolutionize patient care. Different technologies for continuous blood pressure monitoring on normal wards have been suggested, but none is established in routine care. Artificial intelligence can be used to analyze the continuous blood pressure waveform and predict hypotension, and identify distinct endotypes of hypotension.

## Data Availability

Not applicable.

## Abbreviations

HPI-software	hypotension prediction index software
MAP	mean arterial pressure
